# The support needs of families raising children with intellectual disability

**DOI:** 10.4102/ajod.v11i0.952

**Published:** 2022-06-27

**Authors:** Mantji J. Modula

**Affiliations:** 1 Department of Health Studies, Faculty of Humanities, University of South Africa, Pretoria, South Africa; 2 School of Nursing, Faculty of Health Sciences, University of the Free State, Bloemfontein, South Africa

**Keywords:** challenges, child, development, family support, intellectual disability, needs

## Abstract

**Background:**

The deinstitutionalisation of children suffering from intellectual disability (ID) is a global phenomenon. Most families raising such children experience a range of difficulties and require supportive systems to cope with physical, social and mental demands in a home environment.

**Objectives:**

The aim of this study was to explore and describe the support needs provided to families raising children with ID in the Capricorn District of the Limpopo province, South Africa.

**Method:**

In-depth individual interviews and focus group discussions were conducted with 26 families directly affected by the experience of caring for and raising children with ID in Capricorn District of the Limpopo province. Inductive thematic analysis was used to identify, categorise and organise the responses of the participants converted into intelligible statements with the assistance of Atlas. Ti version 8.

**Results:**

Participants identified support needs on information regarding care and management of the children with intellectual disabilities, professional collaboration on safety of the children, community involvement on the rearing of the children and improvement of their living conditions as most of the families and households were female-headed, of low income and needed further monetary support. Overall, the totality of challenges, demands and inadequate support services coalesced in marginalisation of children with ID and their families.

**Conclusion:**

Families raising children with ID are diverse and complex with unique support needs. Therefore, a multilayered approach should be taken to address the concerns and improve the families’ quality of life. A foreseen challenge would be to secure the involvement of the stakeholders representing a variety of sectors, organisations and services.

## Introduction

Intellectual disability (ID) refers to a form of incapacitation that is distinguishable by significant intellectual and adaptive behavioural limitations that emerge before an individual reaches 18 years of age (American Psychiatric Association [APA] 2013; Sadock, Sadock & Ruiz 2015). In this study, the researcher considers ID as referring to mild, moderate, severe or profound cognitive development that stifles a child’s overall growth and development. The social model associates the disability with disabling barriers, societal attitudes and response towards people with impairments. It further focuses on environmental, social and material barriers, such as culture, policies, influences and practices, which cause restrictions, limitations and exclusion of persons with disabilities to participate in the activities of societies (Oliver 1990).

The World Health Organization (WHO) (2011) reported that persons with ID appear more disadvantaged than those presenting with other disabilities. This is because of the long-term physical, neurological, cognitive, sensory and psychological challenges affecting their interaction and functioning in society (United Nations 2006). The WHO (2012) mentioned further that most children with mild and moderate disabilities are unidentified until at school-entry in most developing countries because of different factors including unavailability of mobile units and clinics to provide diagnostic services.

Intellectual disability deficit factors are classified as mild, moderate, severe or profound impairment in accordance with adaptive functioning rather than intelligence quotient (IQ) (Sadock et al. 2015). The South African census of 2011 reported that 4.2% of people presented with memory and concentration impairment (3.2% mild and 1.0% severe difficulties) (Statistics South Africa 2014). According to WHO (2012), lower-income countries encounter higher rates of mild to moderate ID, which is a manifestation of poverty and deprivation that undermine the development of vulnerable children. Furthermore, in these countries, the prevalence rates of people with high numbers of ID were found to be in rural areas across the world (WHO 2011).

Article 7 of the United Nations Convention on the Rights of Persons with Disabilities (UNCRPD) mandates all countries to uphold equal human rights and fundamental freedoms of children with disabilities (United Nations 2006). The promotion of these children’s rights confirms the obligation to protect them from unjust treatment, guaranteed access to health, rehabilitation, education and protection from exploitation (UNICEF 2007). In this regard, the family is obliged to provide care and support to its young, sick and elderly members (DoSD 2013).

In the South African context, the multicultural nature of the society has contributed to the evolvement of types of families and households, ranging from nuclear, single-parent, extended, skip and three-generation households, polygamous, female and child-headed, same-sex, cohabitation and migrant families (DoSD 2013). The White Paper on Families in South Africa has identified that the primary roles of families towards their members were that of fostering membership and family formation, developing economic support, as well as nurturance and socialisation (DoSD 2013). At the same time, the family’s ability to care for the needs of the children with ID is dependent on the competence of the community to meet the family’s diverse support needs (McKenzie 2013). In this regard, the researcher found it significant to conduct this study to explore the support needed by these families living and raising children with ID in South African context.

Statistics South Africa (2014) reported that there were 2 870 130 South Africans (7.5% of the population) living with disabilities in the 2011 census year. For purposes of this study, particular focus would be on those between 5 and 19 years of age as, it was assumed, the children were not recently diagnosed and their families had adequate experience of raising these children with ID. Accordingly, those aged 5–9 years constituted 10.8% (*n* = 447 843) of the population, followed by the 10–14 years’ age cohort (4.1%, *n* = 447 843) and the 15–19 years age group (2.6%, *n* = 108 738). South Africa faces a lack of reliable statistical information of persons with disability and poor tracking systems for recording of service attendance (Mkabile & Swartz 2020), which adversely impacts the ability of the government to make decisive interventions to cater for their needs. The current statistical information on disability relied on the national census. Although foetal alcohol spectrum disorder was found to be the most common cause of ID in South Africa, other factors such as malnutrition, infectious diseases and injuries also contributed to high ID incidences (McKenzie et al. 2019). Deficient statistical information compounds the situation when children continue to be hidden by their families and cannot access any services envisioned in the Integrated National Strategy on Support Services to Children with Disability (INSSSCD) (DoSD 2009).

As the primary caregiver in the life of the child with ID, the family faces their own difficulties in providing normal expected life functions in most cases (Adithyan, Sivakami & John 2017; Pan & Ye 2015). It is evident that families of children with ID experience increased stress levels that threaten the integrity of the family structure (Ahmad & Khanam 2016). As the principal and most permanent support setting to children with ID, the family also deserves supportive services to strengthen normal family life of its members (Irazabal, Pastor & Molina 2016). Nevertheless, the rearing of any child by the family requires support and availability of resources (McKenzie & McConkey 2016). South Africa is one of the upper middle-income countries where the majority of black African families continue to live in poverty and lack of specialised services for ID (Mkabile et al. 2021). Few studies have been conducted on support experiences of families of children with ID in South Africa and little is known about the survival of these families to cater for the needs of their children with ID. The researcher found it important to assess and explore the support needed by these families to provide care and support to their children with ID in the Limpopo province.

In South Africa, Family and Parenting Support Policy indicates that some children are raised by a sequence of primary caregivers, including female relatives of their parents, grandparents or their own generation in families with low monthly income (UNICEF 2015). The Family and Parenting Support policy stresses that the family is a societal unit, which requires ecological balance planted within supportive networks. Hence, support is focused on stability and general functioning of the family (UNICEF 2015). The fundamental goals of family support intended to encourage positive feelings towards the family to commence and continue proactively in taking appropriate steps to raise fulfilled children (Fujioka et al. 2015). It is evident that families of children with ID deserve maximum support (Irazabal et al. 2016), to the extent that further research studies should be undertaken to identify appropriate support services for families (McKenzie & McConkey 2016). However, the support needs of the families raising the children with ID are constantly changing as the children grow up and experience more obstacles in life (Krajnc & Seršen 2017). Therefore, the researcher undertook this study during her PhD studies to understand the experiences and the support needed to enable these families to cope in this continuous changing environment. The study was conducted to explore and describe the support needs provided to families raising children with ID in the Capricorn District of the Limpopo province, South Africa. The specific objectives of the study were:

- to explore and describe the challenges experienced by families raising children with ID
- to assess the existing support programmes and services provided to families living with children with ID
- to propose a range of recommendations for professionals regarding the support needs of the children with ID and their families.

## Research design and methods

### Study design

This study adopted a qualitative research approach to facilitate and enhance a stakeholder-centric mode of data collection, analysis and interpretation (Gray, Grove & Sutherland 2017). This design was most suitable because it enabled a more detailed exploration and description of the ID phenomenon from the participants’ perspectives in an unconstrained manner through focus group discussions and in-depth interviews (Gray et al. 2017).

### Setting

The study was undertaken in the Capricorn District Municipality of the Limpopo province, which comprises the Blouberg, Lepelle-Nkumpi, Molemole and Polokwane municipalities. The Capricorn District Municipality is one of the Limpopo province’s five major municipalities. The other four district municipalities are Mopani, Greater Sekhukhune, Vhembe and Waterberg. The Capricorn District Municipality is approximately 80% rural, with a uniquely diverse ethnic and cultural mix that includes five distinct language groups (Capricorn District Municipality 2017). The district has a higher economic growth potential, compared with the other four provincial districts (Capricorn District Municipality 2017). The district also comprises 30 traditional authorities and is concentrated with a high population density because of the attraction of possible job opportunities, better healthcare and schooling facilities (Capricorn District Municipality 2017). Mankweng and Polokwane tertiary hospitals are located in this district. It is this range of socio-economic and developmental factors that convinced the researcher to undertake the study in this district municipality that is known for better service delivery compared with other municipalities in the Limpopo province to explore and describe the support needs provided to families raising children with ID.

### Study population and sampling

The study population consisted of primary caregiving family members who were available, accessible and willing to share their experiences of raising children living with ID. The vulnerability of the families of children with ID leads some families to hide their children from the community because of the social stigma around the diagnosis. This resulted in the researcher employing the purposive snowballing technique by approaching families whom their children with ID were accessing health, social and education services in their communities. These families introduced other families they knew or met during community gatherings, school meetings and functions to the researcher (Brink, Van der Walt & Van Rensburg, 2018). This approach enabled the researcher to identify even families who were unknown at nearby health facilities and schools.

The eligibility criteria included any member of the families whose specific characteristics were that they had direct caregiving responsibilities, experience and exposure to children living with ID (Polit & Beck 2017). In addition, the researcher’s own judgement was also instrumental in the selection and inclusion of family members whose children were above 6 years of age or of school-going age. At this age most children’s diagnoses of ID are already confirmed as their developmental capabilities were able to be compared with their peers at schools. The included family members were mothers, fathers, grandparents, aunts, uncles, as well as guardians directly involved in raising the children with ID. However, some families are not able to identify the cognitive dysfunction or delayed developments of the children with ID till they are diagnosed at schools. Based on this, it was the researcher’s opinion that these children have not been recently diagnosed and were emotionally ready to express their feelings (not in the study, but generally in their life circumstances). Such families were assumed to have adequate information to share on the lived experience of raising and rearing the children with ID. These factors helped the researcher to include these families to explore and describe their challenges experienced in raising the children with ID. The researcher excluded the families of the children with intellectual disabilities under the age of 6 and above 19 years and those who did not meet the selection criteria. Eventually, 26 volunteering individuals from different families were selected, which comprised 16 mothers, 3 aunts, 2 uncles, 1 father, 1 grandmother, 1 grandfather and 2 guardians of children living with ID.

### Data collection

The researcher conducted 16 individual in-depth interviews and one focus group discussion of 10 members complemented with observational field notes to maximise the data collection process and its anticipated outcomes (Saldana & Omasta 2018). The researcher used field notes to document field-based observations that would not have been captured on the audio recorder during both the interviews and focus group discussions (Marshall & Rossman 2016). Furthermore, the field notes helped the researcher to document pertinent information such as the participants’ emotional and psychological state and attitude towards questions posed to them. The researcher used interview guides designed in a semi-structured format to allow the element of comparability of information from different participants of both the individual in-depth interviews and focus group discussions (Saldana & Omasta 2018). The researcher included different groups of families for both individual in-depth interviews and focus group discussion to ensure richness of data (Lambert & Loiselle 2008). Each individual interview lasted between 45 and 60 min, whilst the focus group discussion endured for about 4 h. The focus group discussion took a long time to enable the families to share their lived experience on the support needed to raise the children with ID. As a result of the length of the focus group, the researcher allowed breaks in between, particularly for parents who brought their children along to attend to their children’s needs. The researcher was cautious of any indications of emotional responses such as fatigue and guarded against exhaustion by focus group members through active engagement during the breaks to encourage participation (Lambert & Loiselle 2008). Both the individual in-depth interviews and focus group discussion were conducted in the home environments where participants raised their children with ID (Polit & Beck 2017). The home environment provided the researcher with better understanding of the families of the children with ID’s real context and the opportunity to reach out to those who were taking care of their children and could not leave their homes.

### Data analysis

The researcher concurrently analysed the data as they were being collected in a continuous, emergent, iterative non-linear process to allow for ongoing reflection, logical questioning and note-taking throughout the study. The researcher listened to the audio-recorded interviews and transcribed each into typed Excel sheet text. The researcher read the transcripts in conjunction with written field notes to acquaint himself or herself with the data (Rubin & Rubin 2012). Various codes were allocated to participants and themes to which each of the participants was associated. The transcripts were uploaded to the Atlas. Ti qualitative data analysis software for a systematic and time-efficient analysis where codes were assigned from an alphanumeric coding list with the assistance of a coding manager. Similar codes were arranged according to emerging ‘families’ of individual and global themes and associated categories and subcategories.

The researcher employed content analysis to arrive at the themes, categories and related subcategories from frequently occurring trends and patterns from the participants’ narrative statements. Data were duly categorised and compared, including examination of any connections, regularities, variations and peculiarities (Rossman & Rallis 2012). Information was summarised into meaningful units, presented into thick descriptions and quotes from the participants to demonstrate their authenticated voice in the context of supporting literature-based evidence (Henning, Van Rensburg & Smit 2013).

### Ethical considerations

The Research Ethics Committee (REC) of the Department of Health Studies, University of South Africa, granted formal permission to the researcher to commence with the study’s empirical data collection process (reference number: HSHDC/860/2018). Both the Limpopo Department of Health (reference number: LP_2018_07_014) and the Capricorn District Senior Manager (reference number: S.5/3/1/2) consequently granted written permission for the study to commence at the study sites under their control. The participants signed an informed consent form as an indication of their formal agreement to participate in the focus group discussion and in-depth individual interviews. All participants gave verbal consent for audio-recording of their narrated statements. The researcher ensured anonymity of the collected data by removing any information that could link the participants to any aspect of the data (Saldana & Omasta 2018).

## Results

The analysed data revealed four thematic responses in terms of support needs pertaining to information, professional, community and improved living conditions for both families raising children with ID and the children themselves.

### Need for information support

The affected families did not receive adequate formal information on ID from health professionals after diagnosis of their children. To that effect, the study found that families lacked basic knowledge regarding the diagnosis and management of the behaviour and overall development of their children with ID. The families have shown the importance of information to understand the care needed to improve the quality life of their family members. The following participant statements testify to this observation by the researcher:

‘The child’s development was up and down. I tried to train the child the way they showed me in the hospital. The child’s development was not well. Everything slowed down. He was able to walk after a long time, maybe after 2 years if I still remember but it took time.’ (Participant 1, 44 years old, mother)

‘The child destroys properties such as curtains and it is difficult to leave her alone. We always make sure that she does not play next to the windows. I do not understand what she sees in them.’ (Participant 11, 27 years old, sister)

‘I delivered in Gauteng Province. I joined a support group. Centurion officers were coming every day in the afternoon to give us information on the condition of my child … I miss that group since I came to Limpopo.’ (Participant 8, 53 years old, mother)

The above excerpts imply that families lack information on ID, including the developmental milestone and understanding the behaviour displayed by children with ID. At the same time, those who had the opportunity to get information in other provinces continue to struggle to access information in the Limpopo province to be able to meet the demands of raising the children with ID.

### Need for professional support

Most participants raised different challenges regarding the support provided by the health professionals and social services. The study revealed poor professional support resulting in poor collaboration between families and professionals providing services to children with ID. The following excerpts bear testimony:

‘When I went to the school to visit my son, I found that he has lost two teeth and the lips were swollen and blue in colour. I was not informed before.’ (Participant 4, 51 years old, mother)

‘They [*professionals*] do not visit us. We do not know them. I just see others visiting those with other conditions including Human Immunodeficiency Virus and Tuberculosis.’ (Participant 5, 63 years old, mother)

‘My child was attending one of the disability schools, but I took the child from school because most of the time they will tell me to come and take the child very often. My child was sent back home by the previous schools without a reason. I am tired of taking my child up and down. I decided that he stays home.’ (Participant 6, 42 years old, mother)

Families blamed professionals (e.g. teachers, nurses, psychologists, speech therapists, social workers and other health professionals) for not informing them about the progress of their children. They further reported poor or irregular home visits by social and healthcare providers. Some reported that they were able to receive support from spiritual leaders. However, the support was dependent on active and regular membership of the family, as evident in the following quote:

‘My pastor was a good counsellor. He was able to visit us when I was still attending church regularly. Currently I am not able to attend church anymore because people became tired of the child’s behaviour, especially because he [*the child*] will just grab things from others. Others cannot tolerate him [*the child*].’ (Participant 11, 27 years old, sister)

### Need for community support

The families indicated that communities lacked understanding of the children with ID. As such, the families displayed the need for structured community support, including that of neighbours, local communities and spiritual organisations. The following statements attest this fact:

‘I have attended support group in the hospital when my child was still admitted. I enjoyed the group and it helped me to know that I was not the only one experiencing difficulties. The social workers were meeting with us every afternoon. I have never attended any since my child was discharged as we don’t have them in our community.’ (Participant 7, 40 years old, mother)

‘She enters every house door and neighbours think that she is a witch because they do not understand her behaviour. People looked surprised by her behaviour.’ (Participant 8, 53 years old, mother)

‘Nothing, no support group. Care centre suggested a support group but parents of children with intellectual disability never attended the meetings. Parents do not seem to be interested. They report that they are always busy.’ (Participant 9, 42 years old, aunt)

The families felt isolated by the community, extended families and friends. Some even decided to withdraw from friends and extended families intending to confront their situations by themselves. Whilst some of the families were aided by support groups, some were not.

### Need for support to improve living conditions

The study revealed lack of resources to support the families’ basic needs and living conditions, which included poor nutrition and housing, lack of sanitation facilities, as well as financial struggles exacerbated by unemployment, especially amongst those mothers who were not working:

‘We cannot find jobs far away because we need to be there for the children. The child’s grant is not enough for his needs. I am not working, the grant for the children I pay R200 for transport of the child, school fees R150 per month and pocket money for children, burial society for the whole family and groceries.’ (Participant 10, 45 years old mother)

‘I must go to their room outside the house to check on him and as a woman I am scared and do not feel safe during the night. He refuses to sleep on the floor in my room. I think the RDP [Rural Development Programme] house will help me to take care of the child during the night. I made application for RDP long time ago and I am still waiting. They follow a list of applicants to build houses and latrines.’ (Participant 11, 27 years old, sister)

‘The school transport fetches him at 08:30 and brings him home at 16:00. It is difficult for me to find work. The reason is that I must look after my child. In most cases, no employer can agree on the employee to work less hours. If I am not home, he [*the child*] goes out of the yard.’ (Participant 12, 43 years old mother)

The accumulated poor living conditions resulted in children not receiving proper education because parents were unable to pay for school transport and the cost of day-care centres. The families further elaborated that the social and disability grants from the government were inadequate and unsustainable. The affected mothers proposed for the government to provide them with caregiver grant because they lost any hope of finding employment to support their families financially.

## Discussion

The findings have shown that the families lack informational support needed to raise the children with ID. This highlights a significant need for various aspects relating to the care and upbringing of children with ID. In this regard, the information need is based on understanding of the child with ID and management of the behaviour of the child, including information on the legal rights of these children to ensure their safety. In addition, information is essential to equip the families with the knowledge to train, care and support their children with ID and further enable these children to learn self-care and basic cognitive skills. However, access to information for self-protection is regarded as a constitutional right for all citizens in South Africa. Furthermore, the White Paper on the Rights of Persons with Disabilities shows that provision of education on ID instills responsibilities to communities and families in caring for and supporting these children (DoSD 2016).

Basically, the social model shows that lack of information by the families to understand ID limit the children’s level of functioning. This finding highlights the support need of the families to receive the adequate information on ID to enhance their understanding on management of these children. In support, the study of Davys, Mitchell and Haigh (2014) found that insufficient knowledge was a barrier to the families to plan for the special care of their children with ID. In addition, the study of Masulani-Mwale et al. (2016) also found that parents of children with ID in Malawi needed to be provided with information on the causes and management of ID. This finding is supported by the study of Krajnc and Seršen (2017), which revealed that parents of children with ID in Slovenia access less information from the services. However, the study of Douglas, Redley & Ottmann (2017) resonated that ID necessitated a quest for knowledge for the parents of the children with ID as the key for reducing stress and adjusting to the condition of the child. Hence, the lack of families in understanding ID in this study compromised the care of the children in their home environment.

In addition, acquisition and assimilation of different types of information is instrumental in directing the nature and type of care for children with ID (Douglas et al. 2017). However, Duma, Tshabalala and Mji (2021) found that the South African families regard the professionals to be the best source of information on planning and managing the care of children with ID. At the same time, supporting the families to understand ID equips them with information to meet the care demands of children with ID. It is in this regard that this study recommends professional educational and training programmes for the affected families in the sphere of knowledge, attitudes, behaviours and skills to encourage and motivate them to participate actively in the care of their children with ID (Caldwell et al. 2018). Furthermore, professionals should provide more in-depth information to the families of children with ID who mostly have less opportunities to self-directed learning (Schmidt, Schmidt & Brown 2017).

Collaboration between professional care providers and the families of children with ID would enhance professional support through mutual respect and communication, whilst also enabling these families to acquire appropriate skills and knowledge concerning the care of their children with ID (Dalmau et al. 2017). However, the findings revealed poor collaboration with families, which created a barrier to communication and resulted in a lack of access to support services. It is this reason that in this study some families preferred to take care of their children on their own to ensure their safety as some children were injured under the care of teachers. In addition, the finding highlights that a safe environment for the children with ID is a struggle irrespective of whether they are at home, day-care centres, schools or community spaces. Similarly, Karisa, McKenzie and De Villiers (2021) found that in Kenya parents took their children with ID to school irrespective of poor safety and care to avoid the $1000 fine or a year’s imprisonment for transgression of compulsory basic education rules for all children. Despite the social model of disability by South African legislation to protect the rights of persons living with ID, children with ID continue to be neglected by societies and the institutions under their care. Although the government developed policies for regulation of educational institutions, including schools and day-care centres, to cater for children with ID, the educators’ role to meet the learning needs is still not aligned with the constitution of the country on educational rights of these children (McKenzie et al. 2019). In addition, the study of Duma et al. (2021) found the need to upskill the teachers on ID to enhance the care of the children with ID and support of their families. The study recommends training programmes for educators, social and healthcare providers to update them on the needs of children with ID and their families.

The findings revealed the need for community support from extended families, friends, neighbours and spiritual organisations to cope with the challenges of raising children with ID. The families felt isolated and not engaged in the community activities compared with those with children without disabilities. Similar to the results of Zechella and Raval (2016), families experienced difficulties in the community or from neighbours who could not understand or accept the condition of the children with ID. The findings were also similar to that of Masulani-Mwale et al. (2016), in Addis Ababa, where the families of children with ID needed support from communities who were excluding them from social activities. In addition, Owen et al. (2017) found that in the United States of America, the availability and access of community-based support services plays an essential role in assisting the families to cope during crisis moments. Some families of children with ID were not willing to attend organised meetings arranged by schools and day-care centres to form support groups. Hence, in this study, home visits by professionals to the families raising these children were essential to encourage and support those who isolate themselves. This finding that is comparable to the study by Wakimizu, Yamaguchi and Fujioka (2018) revealed that home visit services were required to support Japanese families raising children with ID. Furthermore, the available community healthcare workers did not involve or engage with the families of children with ID in community programmes. As such, the families felt neglected by these primary and community-based healthcare service providers.

Some families appreciated belonging to support groups of other families experiencing similar problems to share their experiences and difficulties associated with raising children with ID. This was indicated by the families given the opportunities to attend support groups when their children were hospitalised. These findings were similar to those of Schmidt et al. (2017), who found that parents enjoyed emotional support from other parents of children with ID. This highlights the need for formation of support groups of the families of children with ID within their communities. For example, Aldersey, Turnbull and Turnbull (2016) found that in Democratic Republic of Congo parents of children with ID formed a national association support group registered as non-governmental organisation to meet their support needs. In this regard, the study suggests facilitation and coordination of support groups by professionals to support the families of children with ID.

This study recommends inclusion of families of children with ID in the community health programmes as a mechanism to strengthen their community-based support systems. Furthermore, strengthening of relationships with friends, neighbours and relatives is recommended through awareness of the ID condition. However, the stigma associated with the disability of the child may cause the family to refrain from reporting the condition, which further leads to denial and perpetuates the extant lack of support (UNICEF 2007). Professionals are encouraged to motivate family, neighbours, peers and relatives to enlist in mentorship programmes aimed at improving child-care competencies. At the same time, facilitation of trained community-based networks enhances family support and family life (Zechella & Raval 2016).

The present study was conducted in rural areas with mostly single mothers who headed their households. Most of these mothers were unemployed and found it difficult to meet the financial needs of their families. This resulted in families experiencing poor living conditions linked to sanitation, nutrition, housing and financial constraints. Most of the families suggested monetary support to augment the disability grant that was regarded as not adequate to cater for children’s needs. The South African government has introduced the RDP to provide free houses and sanitation (French drains, ventilated and ordinary pit latrines) to low socio-economic status families. However, some families indicated that the process is slow and does not benefit them. In addition, the government provides independency care grant for the children living with ID. Nevertheless, the families indicated that the grant was inadequate and could not even cater for their elementary needs.

Previous studies have shown that many families in African countries were experiencing low socio-economic status and living in extreme poverty (Keskinova, Cicevska-Jovanova & Ajdinski 2013; Zuurmond et al. 2016). In this regard, the South African government endeavours to continuously provide a supportive environment. For example, the Department of Basic Education in the Limpopo province provides for the free transportation of children with ID to their special schools and free meals for children in need at schools. However, the service does not benefit all the children as the study findings have shown that some families continue to pay school transport for children with ID. This finding is similar to the study of Vergunst et al. (2015) conducted in the Eastern Cape Province of South Africa, where some children were not able to access free transport services and their families paid high costs from their pockets. In this study some parents were not able to pay expensive transport to take their children to school. Consequently, these children were unable to attend school regularly and missed the opportunity to access free meals provided at the school through feeding schemes. Accordingly, the study recommends prioritisation of basic services to families raising children with ID to improve their living conditions. Similarly, the National Strategy on Support Services to Children with Disability of the Department of Social Development has shown that children with intellectual and other forms of disability in the Limpopo province were particularly marginalised and received limited and inequitable support services (DoSD 2009). This further creates a difficult environment for the children to access healthcare, education, rehabilitation and early childhood development services to meet their needs. This study recommends involvement of government departments including that of roads and transport, social, human settlement and treasury to identify and assist families of children with ID in need of support to enable their functioning.

## Limitations and strengths

This study was conducted in the Limpopo province only, with families of children with ID. This could limit the generalisability of the study to other provinces, as well as the views of those families whose children with ID were adequately supported. The study also excluded the families of children who were disabled in other ways than intellectual. However, this study was supported by literature and previous studies and the findings could be transferred to other locations displaying similar characteristics (Brink et al. 2018; Polit & Beck 2017). Furthermore, statistical details and information on the prevalence of children experiencing ID in the Limpopo province and Capricorn District were not available for comparative reference and background information. Single mothers headed most families and most fathers had migrated to cities and other provinces in pursuit of employment opportunities. Collectively, these two factors limited the male voice in the study. However, the experiences and challenges that were shared and repeated by several families contributed to the reliability of the study.

## Implications for research

The outcomes of the study contribute to knowledge in the realm of support recommended for families of children living with ID. For policymaking, this study provides further evidence-based recommendations for government departments to address the support needed by families of children living with ID. Furthermore, it is critical to improve planning and implementation of professional support programmes and systems that significantly enhance the quality of life of the families living with children with ID. More generally, supporting families to address the challenges experienced during the care of children with ID is important at various practical levels. Thus, professionals providing services to children with ID should engage with families of these children on a continuous basis. Such engagement should include feedback on the progress of the children with ID. Although the focus of this study is on family support needs, it is important to understand deinstitutionalisation implications on the families of children with ID.

## Conclusion

This study focused on the support needs of families raising children with ID and made multilayered recommendations to address the current support needs. The study further supports the need for government departments, non-governmental organisations, rehabilitation centres, special schools, day-care centres, local municipalities, community leaders, faith organisations, communities and all relevant stakeholders to participate actively and collaboratively in supporting families raising children with ID for better quality of life.
